# Rehabilitative Interventions and Brain Plasticity in Autism Spectrum Disorders: Focus on MRI-Based Studies

**DOI:** 10.3389/fnins.2016.00139

**Published:** 2016-03-31

**Authors:** Sara Calderoni, Lucia Billeci, Antonio Narzisi, Paolo Brambilla, Alessandra Retico, Filippo Muratori

**Affiliations:** ^1^IRCCS Stella Maris FoundationPisa, Italy; ^2^Department of Clinical and Experimental Medicine, University of PisaPisa, Italy; ^3^Department of Neurosciences and Mental Health, Fondazione IRCCS Ca' Granda Ospedale Maggiore Policlinico, University of MilanMilan, Italy; ^4^Department of Psychiatry and Behavioral Sciences, University of Texas Health Science Center at HoustonHouston, TX, USA; ^5^Pisa Section of National Institute of Nuclear PhysicsPisa, Italy

**Keywords:** autism spectrum disorder, brain plasticity, magnetic resonance imaging, treatment effects, outcome

## Abstract

Clinical and research evidence supports the efficacy of rehabilitative intervention for improving targeted skills or global outcomes in individuals with autism spectrum disorder (ASD). However, putative mechanisms of structural and functional brain changes are poorly understood. This review aims to investigate the research literature on the neural circuit modifications after non-pharmacological intervention. For this purpose, longitudinal studies that used magnetic resonance imaging (MRI)-based techniques at the start and at the end of the trial to evaluate the neural effects of rehabilitative treatment in subjects with ASD were identified. The six included studies involved a limited number of patients in the active group (from 2 to 16), and differed by acquisition method (task-related and resting-state functional MRI) as well as by functional MRI tasks. Overall, the results produced by the selected investigations demonstrated brain plasticity during the treatment interval that results in an activation/functional connectivity more similar to those of subjects with typical development (TD). Repeated MRI evaluation may represent a promising tool for the detection of neural changes in response to treatment in patients with ASD. However, large-scale randomized controlled trials after standardized rehabilitative intervention are required before translating these preliminary results into clinical use.

## Introduction

Autism spectrum disorders (ASD) are a heterogeneous group of neurodevelopmental conditions characterized by persistent deficits in social communication and interaction across multiple contexts, in addition to restricted, repetitive patterns of behavior, interests, or activities, all of which significantly impact on adaptive functioning (American Psychiatric Association, 2013). Although the exact etiology of ASD remains elusive, a combination of genetic and environmental factors during critical periods of development has been implicated (Hallmayer et al., 2011), possibly leading to altered brain architecture beginning early in life (Wolff and Piven, 2013; Conti et al., 2015), or even in prenatal developmental stages (Stoner et al., 2014). Specifically, brain underpinnings revealed by magnetic resonance imaging (MRI) include an early altered developmental trajectory of global and regional brain structures (see Chen et al., 2011 and Bellani et al., 2013 for reviews of structural MRI studies in ASD), with an atypical growing of white matter tracts (see Ameis and Catani, 2015 for a review of diffusion tensor imaging -DTI- studies in ASD), altered task-dependent cerebral response (see Dickstein et al., 2013 for a review of functional MRI -fMRI- studies in ASD), and abnormal neuronal activity in the absence of stimulation (see Uddin et al., 2013 for a review of resting-state fMRI -rs-fMRI- studies in ASD). Notably, abnormalities in brain correlates of ASD are likely to be influenced by study design characteristics and a wide range of clinical and demographic features (Lenroot and Yeung, 2013). In particular, an age-dependency of brain volume differences between ASD patients and controls with typical development (TD) has been repeatedly detected: in fact, while an increased total brain volume is noted in infants and toddlers with ASD compared to TD, an absence of group differences later in childhood is frequently reported (Courchesne et al., 2007; Amaral et al., 2008). The deviation from the normal brain growth trajectory described in young subjects with ASD can lead to atypical organization of structural and functional cerebral connectivity (Lewis and Elman, 2008). Indeed, an excess of short-distance with diminished long-range connectivity has been proposed (Just et al., 2007), producing a brain profile ineffective for processing and integrating “higher-order” information that ultimately leads to several of the most common ASD neuropsychological characteristics (Wass, 2011; Narzisi et al., 2013). In this framework, a rehabilitative intervention for patients with ASD is considered a treatment able to enhance neuroplasticity (Dawson, 2008), i.e., the capacity of cerebral neurons and neural circuits to structurally and functionally change in response to external stimuli, environmental modifications, or injuries (Pascual-Leone et al., 2005). However, neural substrates underlying observed clinical improvement after early interventions are not yet fully elucidate (Sullivan et al., 2014). In this view, the introduction of advanced MRI techniques, such as fMRI, rs-fMRI, and DTI, have been recently used to investigate brain plasticity by monitoring the effects of rehabilitative therapy in ASD patients. Specifically, longitudinal studies that include pre- and post-treatment MRI acquisition have provided new insights on the neural mechanisms targeted in rehabilitative therapy and, in addition, an objective measure of response to treatment.

Therefore, the goal of the current review is to summarize the existing MRI-based evidences of functional and structural plasticity induced by rehabilitation therapy in patients with ASD. To this aim, longitudinal studies that use MRI-based techniques pre- and post- rehabilitative intervention to explore the impact of treatment on neural substrates in ASD patients were analyzed. To our knowledge, no review article exists addressing the question of whether and how non-psychopharmacological interventions shape the brain of patients with ASD.

## Methods

Studies in which functional or structural MRI was used to evaluate rehabilitative treatment response in patients with ASD were eligible for inclusion. Relevant articles were identified from searches in two electronic databases (Pubmed and Scopus). Search terms included the following: “autis^*^,” “neuroimaging,” “MRI,” “magnetic resonance,” “fMRI,” “rs-fMRI,” “DTI,” “diffusion tensor,” “training,” “treatment,” and “rehabilitation” both in isolation and in combination. We further limited the results to “English” and “Humans.” No article type limitations or time period restrictions were applied, and the latest search was undertaken in September 2015. We identified longitudinal studies that examined MRI-based differences in brain structure and function between pre- and post- rehabilitative treatment in individuals with ASD. This paper reports a selective narrative description of the identified investigations.

## Results

We found six studies published between 2006 and 2015 that investigated whether rehabilitation strategies enhance brain plasticity, as evaluated by either rs-fMRI (*n* = 1) (Murdaugh et al., 2015), or task-related fMRI (*n* = 5; Bölte et al., 2006, 2015; Voos et al., 2013; Ventola et al., 2015; Murdaugh et al., 2016). A list of these studies and their characteristics are detailed in Table 1.

**Table 1:** **Summary of studies utilizing MRI pre- and post-rehabilitative treatment of ASD patients**.

	**Bölte et al., 2006**	**Voos et al., 2013**	**Ventola et al., 2015**	**Murdaugh et al., 2015**	**Bölte et al., 2015**	**Murdaugh et al., 2016**
Study type	Randomized cross-over trial	Case series	Uncontrolled pre-post trial	Randomized cross-over trial	Non-randomized parallel group trial	Randomized cross-over trial
Center	Frankfurt University, Germany	Yale University, USA	Yale University, USA	University of Alabama at Birmingham, USA	Frankfurt University, Germany	University of Alabama at Birmingham, USA
Number of ASD patients in the active group	5 males	2 (1 male and 1 female)	10 (8 males)	16 (12 males)	16 males	13 (11 males)
Controls	5 ASD males	No	5 TD (2 males)	15 ASD (12 males) and 22 TD (16 males)	16 ASD (14 males) and 25 TD (21 males)	13 ASD (10 males) and 19 TD (14 males)
Mean age of ASD patients	29.4 years	~5 years	~5 years	10.3 years	19.3 years	10.9 years
ID in ASD patients	No	No	No	No	No	No
MRI technique	fMRI (facial affect recognition task)	fMRI (social perception task)	fMRI (social perception task)	rs-fMRI	fMRI (facial affect recognition task)	fMRI (sentence comprehension task) and rs-fMRI
Type of treatment	Computer-based facial affect recognition training	PRT	PRT	Reading intervention	Computer-based facial affect recognition training	Reading intervention
Duration of treatment	2 h per week for 5 weeks	8–10 h per week for 4 months	7 h per week for 16 weeks	20 h per week for 10 weeks	1 h per week for 8 weeks	20 h per week for 10 weeks
Clinical outcome(s) in the active group	Improvements in facial affect recognition	Improvement on ADOS, CELF-4, and Vineland-II scores	Improvement on SRS-2 and ADOS scores	Improvement in reading comprehension, as measured by the GORT-4 Comprehension subtest	Improvements in facial affect recognition	Improvement in reading comprehension, as measured by the GORT-4 Comprehension subtest
MRI outcome(s) in the active group	Increased activation in the R superior parietal lobule and maintained activation in the R medial occipital gyrus	Different increased activation in the two patients: L dorsolateral prefrontal cortex and L fusiform gyrus in one subject, R posterior superior temporal sulcus, L ventrolateral prefrontal cortex and bilateral fusiform gyri in the other patient	5 ASD patients showed significant increased activation in R posterior superior temporal sulcus, ventral striatum, and putamen; 5 ASD patients showed significant decreased activation in R posterior superior temporal sulcus, thalamus, amygdala, and hippocampus	Enhanced connectivity of Broca's area with R middle frontal gyrus, R superior temporal gyrus, L supramarginal gyrus, and R caudate; reduced connectivity of Broca's area with R middle occipital gyrus and R posterior cingulate cortex	Increased activation in amygdala, fusiform gyrus, and temporal pole bilaterally, medial prefrontal cortex, and L posterior superior temporal sulcus	Increased activation in brain regions underlying language and visuospatial processing, bilateral insula, R postcentral gyrus, and compensatory recruitment of R-hemisphere and subcortical regions
						Increased functional connectivity between L middle temporal gyrus and L frontal regions
					Enhanced connectivity of Wernicke's area with R anterior cingulate, L middle orbital gyrus, bilateral inferior frontal gyrus, R middle frontal gyrus, R middle cingulate, R precentral gyrus	
Follow-up period	No	No	No	No	No	No

*ASD, Autism Spectrum Disorders; MRI, Magnetic Resonance Imaging; DTI, Diffusion Tensor Imaging; fMRI, functional Magnetic Resonance Imaging; rs-fMRI, resting-state functional Magnetic Resonance Imaging; ID, intellectual disability; TD, controls with typical developing; PRT, Pivotal Response Treatment; CELF-4, Clinical Evaluation of Language Fundamentals—Fourth Edition; ADOS, Autism Diagnostic Observation Schedule; SRS-2, Social Responsiveness Scale-Second Edition; GORT-4, Gray Oral Reading Tests-4th Edition; R, right; L, left*.

The first attempt to demonstrate the presence of brain plasticity in ASD following a specific rehabilitation treatment was performed by Bölte et al. (2006) who selected (with randomization) 10 adult patients. Of them, five received a 5-weeks computer-based facial affect training program (2-h per week), while the remaining five ASD patients did not undergo any special training. The effects of training program have been demonstrated on both behavioral and neurobiological level.

However, an atypical pattern of increased activation in the right superior parietal lobule post-training in ASD subjects, rather than the activation of the fusiform face area usually found in participants with TD, was detected. Therefore, the authors concluded that the intervention group learned to recognize emotions by using compensatory mechanisms involving the recruitment of different brain regions.

More recently, a similar study was conducted by the same group (Bölte et al., 2015) to explore the neural effects of the same computer based training (1 h/week for 8 weeks) aimed at improving facial affect recognition. The investigation focused on 32 adolescent with high-functioning ASD: half of them received the specific training plus standard care, whereas the other half received standard care only. Each ASD group was scanned twice, pre and post-training, using task-related fMRI consisting of facial affect recognition. A control group of 25 subjects with TD was also enrolled to compare the behavioral assessment and the brain activations to that of patients with ASD at baseline. As expected, reduced ability to recognize facial expressions coupled with reduced social brain activity was found in individuals with ASD. After training was completed, a relevant improvement in facial recognition characterized the active group and was associated with an increased activity in the social brain areas.

To investigate the possibility that early rehabilitative treatment induces activation changes of brain regions involved in social perception in young children with ASD, Voos et al. (2013) included two ASD preschoolers to receive 8–10 h per week for 4 months of Pivotal Response Treatment (PRT), a well-known empirically validated behavioral treatment for children with ASD (Koegel et al., 1987). After rehabilitation, significant behavioral improvements in core ASD deficits, adaptive skills, and language were signaled and, at the neural level, a modification in the processing of biological motion. Interestingly, the effects of the same treatment on brain activation are different in the two children, in line with the heterogeneity of the response to therapy in the ASD condition (Kim et al., 2015).

The same group performed a subsequent work based on an enlarged sample size of 10 preschoolers with ASD (Ventola et al., 2015). Before treatment, two groups of ASD children could be distinguished based on their activation profiles–reduced or increased- in posterior superior temporal sulcus (pSTS) in comparison with TD controls. Following a 16-week PRT treatment (7-h per week) a significant improvement of clinical ASD manifestations emerged, coupled with an activation in pSTS more similar to that of TD children. Therefore, treatment modifications are reached through two different neural modalities: a decrease in activation following treatment for ASD subjects who exhibited hyper-activation in the pSTS at baseline and *vice-versa*.

A randomized clinical trial (Murdaugh et al., 2015) was designed to explore modifications in functional connectivity after the intensive reading intervention “Visualizing and Verbalizing for Language Comprehension and Thinking” (Lindamood and Bell, 1997) in children with ASD. A total of 31 ASD patients were randomized either to a 10-week reading intervention (active group) or to receive the same intervention after the two imaging sessions were completed (wait-list control group). An additional control group of TD children served as a baseline comparison for brain activation and did not participate in the intervention protocol. Rs-fMRI revealed that after the training the active group had an increased connectivity using Broca's and Wernicke's area as seeding points. In order to verify whether functional connectivity changes in the active group were specifically related to the targeted training, a comparison between post-intervention connectivity in active and control ASD participants was conducted. After rehabilitation, increased rs-fMRI within the reading network was found in the active group, as well as an additional recruitment of frontal regions (left superior frontal gyrus and middle frontal gyrus), interpreted as compensatory mechanisms for language comprehension.

Some subjects originally enrolled in this latter study also underwent fMRI (Murdaugh et al., 2016), in order to investigate changes in brain activation following the reading intervention (Lindamood and Bell, 1997). Pre- and post-training task-related fMRI (sentence comprehension) revealed increased activation in visual and posterior language regions, bilateral insula and right postcentral gyrus, with an additional recruitment of right-hemisphere and subcortical regions as possible compensatory mechanisms for language comprehension. Moreover, an increased functional connectivity between left middle temporal gyrus and left frontal regions has been found after training in the active group. Intriguingly, the intervention-induced brain modifications positively correlated with the improvements in individual reading comprehension. Compared to ASD wait-list control group at the second imaging session, patients in the active group showed an increased activation in some areas of frontal, parietal, and temporal cortices.

## Discussion

Designing studies able to measure neural change in response to therapy is thought to be an important goal for autism research (McPartland and Pelphrey, 2012). According to this view, the current review investigated studies that applied MRI-based neuroimaging techniques pre- and post-rehabilitative treatment in ASD. Results from the six included investigations (Bölte et al., 2006, 2015; Voos et al., 2013; Murdaugh et al., 2015, 2016; Ventola et al., 2015) suggested that training-induced behavioral changes were accompanied by significant modifications in neural activity and/or functional connectivity that varied as a function of the specific training intervention. Preliminary evidence suggests that early intervention can mitigate the severity of core and associated features of autism (Warren et al., 2011), improve the long-term outcome of treated patients (Estes et al., 2015), and even reverse some of the ASD symptoms (Rogers et al., 2014). These behavioral improvements are supposed to result from changes in brain structure and function that are particularly achievable in critical period plasticity in early life. At these specific time windows, environmental stimuli most potently shape cortical brain circuitries responsible for the acquisition of different types of skills and abilities (Bardin, 2012). However, results from the studies included in this review reflect that brain plasticity, also in ASD patients, is not limited to early developmental stage (Voos et al., 2013; Ventola et al., 2015), but includes also the school-age period (Murdaugh et al., 2015, 2016), and adulthood (Bölte et al., 2006, 2015).

Notably, the six included studies pertained to three research group, each of whom performed two investigations to evaluate the effects of a specific rehabilitative treatment. In this context, rehabilitation treatments received by patients with ASD are highly heterogeneous in terms of targeted impairments. In particular, the group coordinated by Ventola focused on the neural effects of a comprehensive intervention program specifically developed to target the core social and communication deficits of children with ASD (Voos et al., 2013; Ventola et al., 2015); Bölte and colleagues utilized a computer-based program to target specific impairment in facial affect recognition (Bölte et al., 2006, 2015), while Murdaugh et al. administered a protocol to improve reading comprehension, an ancillary deficit in ASD individuals (Murdaugh et al., 2015, 2016).

In spite of the encouraging results on behavioral and brain plasticity, the studies examined in this review suffer from several drawbacks. In fact, reports generally comprised of a limited number of subjects (ranging from 2 to 16 ASD individuals in the active group), and the relatively small sample size makes it difficult to homogeneously subgroup ASD patients based on their clinical profile (e.g., level of intelligence quotient and language, core ASD symptom severity, adaptive functioning, psychiatric comorbidities). Therefore, it is difficult to identify baseline clinical characteristics that influence behavioral and brain outcome of examined patients. Moreover, the lack of MRI follow-up data after the second scan hampered testing for stability of brain modifications obtained following rehabilitative treatment. In fact, it remains to investigate whether such improvements in brain functions are sustained without further intervention or whether maintenance training is necessary.

In addition, the studies included in the current review had different design. Two investigations (Voos et al., 2013; Ventola et al., 2015) lacked of a non-active control group of ASD subjects, whereas the others included as control group subjects in the waitlist (Bölte et al., 2006; Murdaugh et al., 2015, 2016), or patients receiving standard care only (Bölte et al., 2015). The presence of a non-active control group of ASD individuals is essential to discern whether an hypothesized biomarker for the prediction of treatment response is really predictive of response to a specific intervention, or rather is predictive of prognosis, independent from the type of rehabilitative treatment or even in the absence of it. Four studies included TD controls who underwent one MRI session (Bölte et al., 2015; Murdaugh et al., 2015, 2016; Ventola et al., 2015) in order to compare brain profiles of ASD patients and controls at baseline. Unfortunately, no study included MRI measures of TD subjects after the treatment under investigation: the lack of these data do not allow to provide evidence that neural changes are ascribable to effects of treatment rather than to normal brain maturation.

Despite their heterogeneity, all the six included studies reported significant modifications in task-related brain activation or in functional connectivity following rehabilitative intervention: crucially, the absence of negative or inconclusive results suggests the risk of publication bias. Instead, it could be useful the clinical and neural characterization of “non-responders” to a specific rehabilitative treatment in order to redirect these patients toward different intervention strategies.

Of particular interest, participants in these studies are generally highly selected individuals (e.g., exclusion of younger patients, and/or subjects with intellectual disability), who differ from patients seen in the clinical practice. Even if this choice is surely motivated by the necessity of patient compliance with the MRI examination (capacity of lying still in a confined space for a long time, as well as of tolerating the acoustic noise and of understanding task instructions), it limited information about brain plasticity in the full range of the autistic spectrum. Future naturalistic studies in larger samples could contribute to identifying biomarkers sensitive to rehabilitative treatment in the conventional clinical population. In this context, the use of sleep MRI (Pierce, 2011), and of task-free MRI techniques (DTI, rs-fMRI) would aid inclusion of non-collaborative ASD patients.

### Future directions

Prognostic factors of effective rehabilitative intervention in ASD are to date poorly clarified and generally based on patient's clinical characteristics and family profile only (Vivanti et al., 2014). The inclusion of MRI-based measures pre- and post-treatment will be crucial for understanding the neural mechanisms underlying different patient outcome. In other words, specific brain regions/network as putative biomarkers for treatment response would be identified, contributing to the knowledge on “what works for whom and why,” and thus paving the way for the individualization of treatment in ASD. In order to overcome this limitation, future randomized control trials should incorporate pre and post-treatment neuroimaging protocols and compare the effects of distinct therapeutic treatments on patient's baseline neural biomarker.

Future investigations would also benefit from machine learning classification techniques to predict response to rehabilitative treatment at an individual level. In these studies, subjects are split into responders and non-responders after treatment, and machine-learning techniques are used to allocate patients to either category based on their pretreatment brain profile. However, the accuracy of these methods is currently not sufficiently satisfactory to suggest their use in clinical practice (Retico et al., 2014; Wolfers et al., 2015).

Finally, it will be crucial the use of multimodal neuroimaging techniques for detecting brain change between baseline and post-training in ASD. In this perspective, some recent studies have investigated connectivity in ASD using simultaneous structural -DTI- and functional -fcMRI- methods (Kana et al., 2012; Delmonte et al., 2013; Deshpande et al., 2013; Mueller et al., 2013; Nair et al., 2013): the integration of these data with neurophysiological approaches (electroencephalography–EEG- and magnetoencephalography–MEG-) may significantly enhance their temporal resolution. Moreover, the combination of both structural (MRI/DTI) and functional (fMRI/EEG) neuroimaging techniques could provide new insights on the timecourse by which the neural changes occur after rehabilitation: preliminary results on healthy older adults suggest that functional change may precede structural and cognitive change (Lampit et al., 2015), but the temporal dynamics of training-induced neural modifications have not been investigated yet in ASD subjects. In this perspective, the detection of functional changes could suggest that the rehabilitative intervention is effective, even if clinical modifications are not yet perceptible in the ASD patient. Thus, it would be possible to prevent dropout from a potentially beneficial intervention and consequently to reduce waste of resources as well as of valuable time for improving ASD prognosis.

## Author contributions

SC wrote the paper with contributions from LB, AN, PB, AR, and FM. All authors read and approved the final manuscript.

### Conflict of interest statement

The authors declare that the research was conducted in the absence of any commercial or financial relationships that could be construed as a potential conflict of interest.
